# Does the Loss of Teeth Have an Impact on Geriatric Patients’ Cognitive Status?

**DOI:** 10.3390/jcm12062328

**Published:** 2023-03-16

**Authors:** Dana Gabriela Budală, Carina Balcoș, Adina Armencia, Dragoș Ioan Virvescu, Costin Iulian Lupu, Elena Raluca Baciu, Roxana Ionela Vasluianu, Monica Tatarciuc, Ionuț Luchian

**Affiliations:** 1 Department of Implantology, Removable Prostheses, Dental Prostheses Technology, Faculty of Dental Medicine, “Grigore T. Popa” University of Medicine and Pharmacy, 700115 Iasi, Romania; 2 Department of Surgery, Faculty of Dental Medicine, “Grigore T. Popa” University of Medicine and Pharmacy, 700115 Iasi, Romania; 3 Department of Odontology-Periodontology, Fixed Restorations, Faculty of Dental Medicine, “Grigore T. Popa” University of Medicine and Pharmacy, 700115 Iasi, Romania

**Keywords:** brain, cognitive impairment, denture, dementia, geriatrics

## Abstract

Significant changes in the microstructure of the brain cause dementia and other mental declines associated with aging and disease. Although research has established a connection between oral health and dementia, the underlying pathologic mechanisms are still unknown. Aim: Our aim was to evaluate dentures’ impact on the cognitive state of geriatric patients. Material and methods: A total of 108 individuals seeking treatment at the Faculty of Dental Medicine in Iasi, Romania, participated in the study, which ran from May 2022 to October 2022. Cognitive dysfunction was assessed using the Mini-Mental State Examination. The acquired data were analyzed with IBM SPSS 26.0, and the *p*-value was set at 0.05. Results: The average value of the MMSE score was 21.81 ± 3.872. Differences between groups of wearer/non-wearer subjects were statistically significant for most of the questions in the questionnaire. Linear regression analysis showed that individuals with a high MMSE score have prosthodontic treatment. A decrease in the MMSE score is associated with a decrease in masticatory efficiency (B = 1.513, *p* = 0.268). Conclusions: This study provides further evidence that tooth loss is associated with worse cognitive performance. It is thus conceivable that the necessary effects can be achieved by increasing the efforts dedicated to preventing tooth loss in the adult population.

## 1. Introduction

The most important trend of the 21st century is the general trend of an aging population. The aging of the population is the outcome of several interrelated changes, including falling birthrates, longer life expectancies, and later deaths [1,2]. By 2050, it is predicted that around 16% of the world’s population will be over the age of 65, which is more than double the present number and a fivefold rise since 1950 [3,4].

Age-related declines in cognitive abilities such as memory, judgment, language, and focus are a natural consequence of the aging process. Neurodegenerative, vascular, and dysthymia/dysphoria disorders are all potential causes. Social, functional, and vocational activities can all be impacted by impairments in cognition and IQ [5,6].

The brain’s microstructure undergoes considerable alterations as a result of aging and diseases, leading to cognitive loss. Changes in brain morphology (the shape and structure of the brain) are a normal part of the aging process, with the most common alteration being significant atrophy [7]. 

The neuroimaging community has extensively examined age- and disease-related changes in the structure of the brain. For the first time, cross-sectional data may be compared directly to form attributes from atlases that are universally acknowledged [8]. Global brain shrinkage, changes in brain functional responses, and cognitive decline are all common side effects of normal aging [8]. As a result of this, brain changes exhibit a significant degree of individual variation and appear to be reliant upon different factors, such as mastication [9].

Impaired cognitive function is associated with both nonmodifiable (such as age and gender) and controllable (such as blood pressure and diabetes) risk factors [10,11,12]. Cognitive impairment is not an illness but a description of a condition. It means that the person in question has trouble with tasks such as memory or paying attention. They might have trouble speaking or understanding. Additionally, they might have difficulty recognizing people, places or things, and might find new places or situations overwhelming. Despite extensive research, no definitive treatment for this cognitive deficit has yet been found [13]. Since more people with mild cognitive impairment than without it go on to acquire Alzheimer’s disease or similar dementia conditions, researchers have tried to examine and prevent mild cognitive impairment in an effort to diminish the societal and financial costs related to the condition. Potentially modifiable risk factors for cognitive impairment have been identified as poor dental health and poor mastication [14].

According to scientific evidence, frequent sensory input when chewing causes an increase in blood flow to the brain and a greater number of pyramidal neurons in the hippocampus. When it comes to humans, this area of the brain is critical for the generation and retrieval of episodic memory [15,16]. Neurotransmitter function may be negatively affected by insufficient mastication capacity as well as by the absence of afferent stimulation by masticatory receptors. This may result in a decrease in the amount of acetylcholine produced, which is responsible for the stimulation of electrical flow between neurons [17,18].

Poor oral health has been associated with cognitive impairment in several long-term cohort studies [19,20]. A correlation has been shown between the number of teeth in a person’s mouth and their level of cognitive performance. It has also been shown in several case studies that restoring tooth and masticatory function with an appropriate prosthesis can increase functional activity in the brain [21,22]. The periodontal ligament and masticatory muscle are thought to receive their nerve supply from the trigeminal nerve. The attenuation of trigeminal nerve sensory input as a result of ongoing tooth loss has been demonstrated to impair higher-level brain functions including learning and memory. [23,24]. Improvements in oral motor performance and shifts in mandibular position are closely connected to deterioration in masticatory muscle function, and degradation of the ά-γ coupling mechanism may be associated with senile dementia in some cases [25]. Previous research has shown a link between dental health and dementia, but the pathogenic processes by which this occurs remain unclear.

Despite the fact that we are aware of certain data that suggest otherwise, we are not aware of any meaningful evidence about the impact that dentures play in the cognitive state of older people who are edentate.

It is becoming increasingly clear that oral health may play a crucial role in a person’s cognitive performance as they age. Many studies have found a link between the number of natural teeth a person has and their cognitive abilities [26,27]. Even if we are aware of certain statistics that suggest otherwise, we are not aware of any meaningful evidence about the impact that dentures play on the cognitive state of older people who have lost all of their teeth.

Although preliminary clinical investigations have supported this logic, it is still just conjectured as to whether or not it is possible to reverse decrease in cognitive function by improving chewing performance through restorative treatments. As a result, the present study was designed to test the hypothesis that dentures, acting through the mastication route, will have an impact on the cognitive state of the senior elderly population.

## 2. Materials and Methods

### 2.1. Study Population

The study was conducted with the approval of the Ethics Committee of the Grigore T. Popa University of Medicine and Pharmacy of Iasi (No. 18/05.05.2022), and the included participants all consented to the procedures. The research was conducted from May 2022 to October 2022. At the recruitment stage, the study objectives were explained, inviting all adults aged 60 years and above to participate in the study. The exclusion criteria were: (1) younger than 60 years old, and (2) cognitive disease already being treated.

Patients seeking treatment at the Faculty of Dental Medicine in Iasi, Romania, were eligible for enrolment; a total of 112 patients who agreed to take part in this study were included.

The intraoral examination was performed by a single examiner who only considered the number of missing teeth and not the efficacy of treatment for determining the edentulousness type. The patients were given free rein as to how they wanted their mastication assessed, and the goal was to determine whether or not cognitive impairment was inversely proportionate to the number of patients with at least some of their original teeth.

### 2.2. Cognitive Dysfunction Assessment

Cognitive dysfunction was assessed using the Mini-Mental State Examination (MMSE), which is a commonly used tool for measuring cognitive function. The MMSE works well as a screening tool to distinguish between patients with and without cognitive impairment. Since its initial publication in 1975, Folstein’s study has been cited about 50,000 times in the Scopus database [28]. Its rapid implementation and widespread use may have contributed to this effect. Furthermore, a recent meta-analysis demonstrated that the instrument’s sensitivity was 85%, with specificity around 90%, for the diagnosis of dementia in both community and primary care settings [29].

The instrument can also measure changes in cognitive status that may benefit from intervention when administered repeatedly. The measure should not take the place of a thorough clinical evaluation of mental status, however, as it is unable to diagnose the circumstances surrounding changes in cognitive function. The test also significantly emphasizes verbal response, reading, and writing.

The Mini-Mental State Examination (MMSE) is used for conducting a complete and methodical evaluation of mental status. The MMSE was translated and validated in Romania [30]. Five cognitive processes are examined in this 11-question test: orientation, registration, attention and calculation, recall, and language. The maximum score achievable is 30. Cognitive impairment is indicated by a score of 23 or less. The MMSE can be administered in just 5–10 min, making it convenient to use frequently and on a regular basis.

### 2.3. Assessment of Covariates

People aged 60 and above have varying degrees of cognitive impairment. There is a wide variety of potential causes, and often these factors overlap. Recent epidemiological studies estimate that between 4.7% and 8.7% of the older population may have dementia, while as many as 42% may be living with moderate cognitive impairment (MCI) [31,32,33]. Controlling for demographic covariates such as age, education, race, and neighborhood (or place of residence) will strengthen the study design.

Screening tests are advised for the diagnosis of cognitive impairment in persons who have a high suspicion of having Alzheimer’s disease (AD) or other disorders. Therefore, we collected information about participants’ sociodemographic characteristics (i.e., age, gender, education level, place of residence) and health conditions (e.g., presence of chronic conditions).

### 2.4. Statistical Analysis

The acquired data were examined with IBM SPSS 26.0 (SPSS Inc. Chicago, IL, USA). The statistical significance was set at *p* = 0.05. The descriptive study of the group’s general characteristics was reported as frequencies, means, and standard deviations. We employed the Student’s *t*-test and the ANOVA test for comparisons. Correlations between MMSE scores and specific variables were determined by applying linear regression (sex, education, edentulous treatment, and masticatory efficiency).

## 3. Results

In total, 108 subjects participated in the study, with an average age of 67.79 ± 14.44 (minimum age of 28 and maximum age of 87) and a greater proportion of female subjects (57.4%), with 53.7% having a high school education, and the majority coming from an urban environment (64.8%). A total of 85.2% of the patients reported comorbidities, with cardiovascular, metabolic, and locomotor problems being the most prevalent (Table 1).

Concerning dentition-related traits, more than half of the participants had several types of edentation (51.9%), followed by those with complete edentation (35.2%). Only 50% of the edentulous participants underwent prosthetic treatments, 25.9% of them with removable dentures and 24.1% having fixed and removable prostheses. Only 46.3% of the individuals demonstrated adequate masticatory efficiency (Table 1).

In Table 2, the distribution of the participants’ answers to the questions of the MMSE questionnaire is presented. The average value of the MMSE score is 21.81 ± 3.872 out of a maximum value of 30. The increased frequency of answers with low scores can be observed in the case of subjects who have teeth not treated with prosthodontic treatment; thus, in the case where the subject had to count backwards from 100 by decreasing by 7, it was observed that 22% of those who did not have prostheses did not have the ability to achieve this, followed by 62% who managed to do this to a small extent.

More than half of the non-wearer subjects had reduced ability to recall the names of three previously heard objects as well as to write a phrase with a subject and a predicate (q 5: score 1–51.9%, q 6: score 0–51.9%). Reproducing a drawing was another situation in which 55.6% of the non-wearer subjects encountered difficulties. Differences between groups of wearer/non-wearer subjects were statistically significant for most of the questions in the questionnaire (Table 2).

The average value of the MMSE score was 21.81 (SD 3.872) and was associated with edentation treatment (*p* = 0.000), subjective masticatory efficiency (*p* = 0.000), and detected comorbidities (*p* = 0.000). There were no associations between the MMSE and gender distribution, education level, or place of origin (Table 3).

Regarding the MMSE forms, depending on the general characteristics, the statistical analysis indicates that female subjects present a higher frequency of moderate MMSE form scores (35.5%), regardless of the level of education, and the mildest MMSE form scores. More subjects with university degrees (75%) and those from the urban environment present more cases of moderate MMSE than those from the rural environment (34.3%).

Statistically significant differences were recorded in the case of the edentulous treatment variables, where non-wearer subjects presented more moderate MMSE (63%, *p* = 0.000), as well as in the case of subjects who declared that they had ineffective mastication (58.6%, *p* = 0.000) and in the case of those who have comorbidities (34.1%, *p* = 0.000) (Table 3).

**Figure 1:**
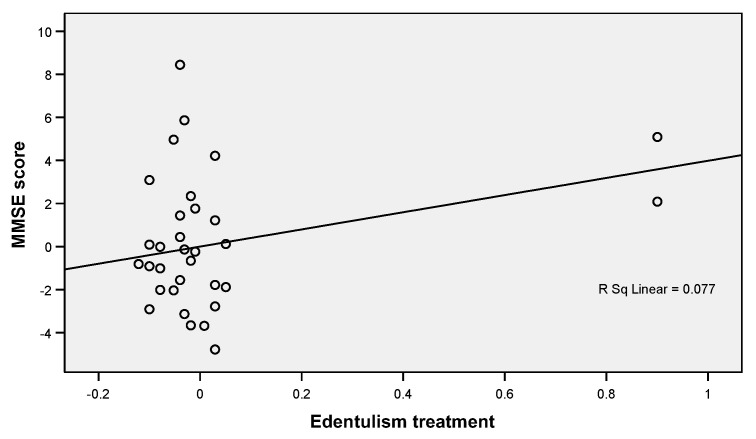
Linear regression plot showing a positive correlation between MMSE scores and edentulous treatments.

Linear regression analysis (Table 4) showed that in the case of the association of the MMSE score and the edentation treatment, the correlation coefficient is positive (B = 3.986, *p* = 0.004), which indicates that individuals with a high MMSE score (close to wave max. 30) have prosthodontic treatment. This relationship is also highlighted in Figure 1, where the regression line is positive and the points are grouped in quadrants I and III, demonstrating a tight, positive, and balanced relationship between the two elements.

**Figure 2:**
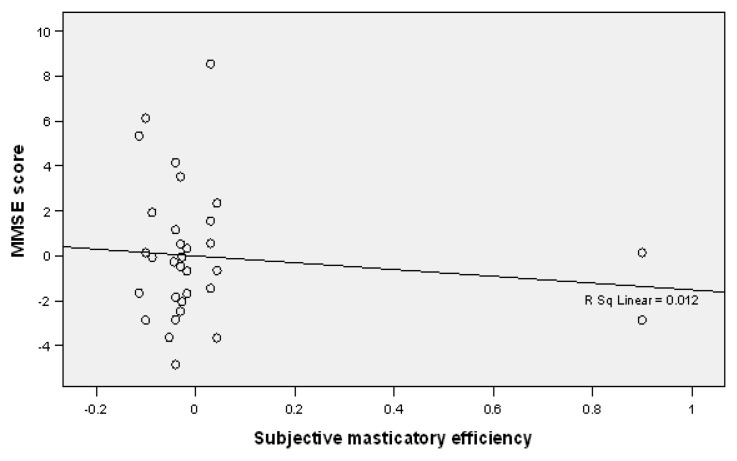
Linear regression plot showing a negative correlation between MMSE scores and subjective masticatory efficiency.

A negative relationship was detected between the MMSE scores and subjective masticatory efficiency, which indicates that a decrease in the MMSE score is accompanied by a decrease in subjectively evaluated masticatory efficiency in the study participants (B = 1.513, *p* = 0.268) (Table 4, Figure 2).

## 4. Discussions

According to recent findings in oral health and geriatric medicine, a new dimension has emerged in the study of significant links between impaired oral function, occlusal/mastication, and specific systemic illnesses such as cognitive and brain functions. Geriatric syndromes, such as memory and cognitive impairments and dementia, can lead to a steady deterioration, which is sometimes accompanied by other comorbidities [34,35].

Cognitive issues are typically more prevalent and disabling in older people as a result of their advanced age. According to the findings of our study, older age was substantially related to the onset of cognitive impairment. This finding was in line with our expectations.

It has been widely debated in the literature [36] whether there is a correlation between one’s socioeconomic position and access to dental treatment, and this does appear to be an essential role in cognitive impairment. In our study, likely due to the limited number of subjects, we did not find any link between the patients’ socioeconomic status and cognitive impairment.

According to several studies, the existence of natural teeth in humans appears to be linked to higher cognitive performance [37,38]. After conducting a literature review on the relationship between occlusion and human brain function, Okamoto and his colleagues [39] came to the conclusion that “mastication and other movements stimulate activity in the cerebral cortex and could be useful in avoiding degeneration of cognitive ability. It has been hypothesized that rhythmic chewing motions, which enhance blood flow in the brain and stimulate various sections of the cortex, are responsible for this phenomenon and that an increase in blood oxygen levels in the prefrontal cortex, as well as in the hippocampus, may influence learning and memory function [40,41].

Losses in masticatory function, rather than number of teeth, has been found to have a significant effect on cognitive performance [42]. In this context, it is well known that the molars are the teeth that can withstand a greater amount of masticatory power and are the primary determinants of masticatory efficiency [43], and this is true for both natural and artificial occlusion. Therefore, masticatory performance can have a favourable influence on cognitive function [44], regardless of whether it is performed with natural teeth or with prosthetic therapy. The results of our study are similar to those of previous studies in the sense that persons with edentulous arches have reduced masticatory efficiency and low MMSE values.

Even more intriguing is the finding that the only significant link between cognitive deterioration and tooth loss was discovered when molars were missing from the mouth after each kind of lost tooth was examined separately. This may be transmitted through the locus coeruleus, which is triggered by a variety of factors including periodontal fibers and proprioceptive jaw muscle spindles [45]. For our study, this could be considered one limitation because we conducted the analysis taking into consideration only the type of edentation and not the type of the remaining teeth.

It is undeniable that tooth loss has been associated with the development of memory and cognitive impairment as well as dementia. Evidence suggests that having fewer than 20 teeth increases the likelihood of cognitive impairment and dementia in the elderly [46,47].

The findings of Shimazaki et al. revealed that around 50% of the entirely edentulous and 35% of the partially edentulous who did not wear dentures acquired over time a considerable risk of physical handicap and death [48].

Therefore, preserving as many natural teeth as possible or wearing dentures that are well fitted can be a vital precaution for the oral and physical health of the elderly, especially the more vulnerable population [49].

Greater chewing capacity from more functional tooth units on dental occlusion may lead to extended life expectancy; similarly, a greater selection of nutrients in daily meals is similar to intellectual and social activities for a higher functional quality of life [50]. According to the findings of recent investigations, age-related oral deafferentation and age-related changes in brain activity might result in cross-modal problems, such as loss of the ability to taste and smell food. Because of animal research in which hard food was used as feed, the relationship between oral deafferentation and the neurocognitive and neurogenic brain axis was further established [51].

Consequently, there has been a rise in the belief that dental deafferentation and brain aging are linked, which might lead to new treatments for cognitive decline and neurodegenerative illness in the elderly. In both humans and animals, the reduction in hippocampal brain-derived neurotrophic factor levels is linked to the deterioration of brain and masticatory processes. At the same time, the number of dendritic spines in molar-less mice with the reduced distinction of newly neuronal resulted cells is inhibited, which may be associated with damage in hippocampus-dependent spatial memory, a decrease in the growth and survival of new-born cells in the dentate gyrus, an increase in hippocampal amyloid-beta, and a deterioration of norepinephrine neurons in the locus coeruleus [51].

Despite the fact that our brain is in a permanent state of flux, new connections are always being formed, which might result in the acquisition of new abilities or adjustment to a new oral environment. More research is needed to determine if the concept of “neuroplasticity” is correct. Studies have also demonstrated the distinctive and reversible neuroplasticity of corticomotor excitability in the context of controlling peri-oral tongue muscles during movements. Two weeks following tongue training, it has been demonstrated that the plastic alterations returned to baseline levels.

A study by Kumar et al. found that in their study group, denture users’ cerebral activity returned to baseline levels three months following the placement of new dentures, which was similar to the results of previous research. This is a return to the starting point [51]. It is possible that cortical modifications were more “elastic” (i.e., reversible) than “plastic” once the training was discontinued, giving the appearance that the changes were more permanent (i.e., irreversible).

The correlation between tooth loss and decreased cognitive performance is supported by the findings of this study. As a result, it is likely that by increasing the efforts that are committed to preventing tooth loss in the adult population it will be possible to achieve the desired results.

It is important to note that this evaluation does have certain limitations. As a result of the small sample size, we were unable to obtain reliable estimations of the parameters governing the study’s validity. We also acknowledge that our participants were sampled from one area of the city of Iasi, which may raise questions regarding the generalizability of our results; a future population study could me more randomized, apart from age and presence/absence of teeth.

## 5. Conclusions and Perspectives

These findings further highlight the positive impact of periodontal medicine and preserving natural teeth on memory. Beyond the financial repercussions, the true cost of cognitive decline, if we define it as memory impairment as well as personal experiences and relationships, is unquantifiable.

As a future perspective for this pilot study, one might convene a multicenter study group, representative for at least a region of Romania and presenting a possible corelation between patients’ cognitive state of mind, prosthetic condition, and quality of life.

Furthermore, it may be possible to retain and safeguard other aspects of a person’s wellbeing that cannot be quantified by preventing tooth loss, such as the capacity to live a comfortable life, the conservation of memories, and the maintenance of a sense of one’s own personality.

## Figures and Tables

**Table 1:** Characteristics of the study participants.

		No.	%
Age	67.79 year (SD 14.44), (min. 28 years, max. 87 years)		
Sex	Female	62	57.4
	Male	46	42.6
Education level	Elementary school	38	35.2
	Secondary school	58	53.7
	University studies	12	11.1
Place of residence	Urban	70	64.8
	Rural	38	35.2
Type of edentation	Partially extended edentulism	14	13.0
	Total edentulism	38	35.2
	Combined edentulism	56	51.9
Treatment of dental edentation	Yes	54	50.0
	No	54	50.0
Type of treatment of dental edentation	Untreated edentulism	54	50.0
	Removable prosthesis	28	25.9
	Composite prosthesis	26	24.1
Subjective masticatory efficiency	Adequate mastication	50	46.3
	Inadequate mastication	58	53.7
Comorbidities	Yes	92	85.2
	No	16	14.8

**Table 2:** Distribution of participants’ answers to the questions of the MMSE questionnaire.

Questions	Mean ± SD Maxim Value	MMSE Scores	Edentulism Treatment		*p*
			No	Yes	
1. Orientation: Which (year), (season), (day of the week), (date), (month) is it?	4.15 ± 0.873	2 3 4 5	18.5% 7.4% 63.0% 11.1%	0.0% 0.0% 37.0% 63.0%	0.000
	Max. 5				
Where are we—(country), (town), (district), (hospital), (floor)?	4.35 ± 0.701	3 4 5	25.9% 51.9% 22.2%	0.0% 25.9% 74.1%	0.000
	Max. 5				
2. Memory: Say the names of three unrelated objects loudly and clearly, with one-second pauses between them. Ask the patient to repeat all three (1 point for each correct answer). If it does not work the first time, repeat the test until the patient repeats all three words (try up to 5 times). If the patient cannot learn them all, immediate memory cannot be properly assessed	2.28 ± 0.653	1 2 3	7.4% 59.3% 33.3%	14.8% 40.7% 44.4%	0.133
	Max. 3				
3. Attention and calculation Subtract 7 from 100, then repeat from the result. Continue five times: 100, 93, 86, 79, 72, 65	2.65 ± 1.328	0 1 2 3 4 5	22.2% 3.7% 33.3% 29.6% 7.4% 3.7%	0.0% 0.0% 25.9% 40.7% 18.5% 14.8%	0.001
	Max. 5				
4. Recall Ask for the names of the three objects learned earlier (1 point for each correct answer).	1.98 ± 0.875	1 2 3	51.9% 18.5% 29.6%	25.9% 29.6% 44.4%	0.022
	Max. 3				
5. Language: “Make up and write a sentence about anything.” (This sentence must contain a noun and a verb.)	1 ± 0.820	0 1 2	51.9% 29.6% 18.5%	14.8% 37.0% 48.1%	0.000
	Max. 2				
Repeat “No ifs, and or buts.”	1 ± 0.00	1	100%	100%	-
	Max. 1				
Follow a 3-stage command: “Take the paper in your right hand, fold it in half, and put it on the floor.” (The examiner gives the patient a piece of blank paper.) Score 1 for each stage.	2.41 ± 0.737	1 2 3	18.5% 33.3% 48.1%	11.1% 25.9% 63.0%	0.277
	Max. 3				
Read and obey the following: “Please read this and do what it says.” (Written instruction is “Close your eyes.”)	0.85 ± 0.35	0 1	29.6% 70.4%	0.0% 100%	0.000
	Max. 1				
Name a pencil and watch.	0.94 ± 0.23	0 1	11.1% 88.9%	0.0% 100%	0.012
	Max. 1				
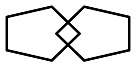 6. Copying:	0.52 ± 0.50	0 1	55.6% 44.4%	40.7% 59.3%	0.177
	Max. 1				
SCOR MMSE	21.81 ± 3.872	16 17 18 19 21 22 23 24 25 27 29 30	22.2% 3.7% 14.8% 33.3% 3.7% 18.5% 0.0% 0.0% 0.0% 0.0% 3.7% 0.0%	0.0% 0.0% 0.0% 0.0% 11.1% 0.0% 40.7% 11.1% 14.8% 7.4% 0.0% 14.8%	0.000
	Max. 30				

**Table 3:** Cognitive impairment: means values for general characteristics, edentulism treatment, and masticatory function.

		Cognitive Impairment						
		Mean	SD	*p* *	No MMSE %	MMSE Mild Form %	MMSE Moderate Form %	*p* **
Sex	Female Male	21.10 22.78	3.696 3.932	0.025	6.5 8.7	58.1 65.2	35.5 26.1	0.565
Education level	Elementary school Secondary school University studies	21.16 21.90 23.50	3.309 4.233 3.398	0.184	0.0 10.3 16.7	65.8 55.2 75.0	34.2 34.5 8.3	0.091
Place of residence	Urban Rural	22.09 21.32	3.907 3.807	0.326	8.6 5.3	57.1 68.4	34.3 26.3	0.503
Treatment of dental edentation	No Yes	19.11 24.52	2.820 2.725	0.000	0.0 14.8	37.0 85.2	63.0 0.0	0.000 *
Subjective masticatory efficiency	Adequate mastication Inadequate mastication	24.68 19.34	2.736 2.881	0.000	16.0 0.0	84.0 41.4	0.0 58.6	0.000 *
Comorbidities	No Yes	24.60 21.18	5.051 3.268	0.000	40.0 0.0	40.0 65.9	20.0 34.1	0.000 *

* *t* test; ** Chi-square test.

**Table 4:** Linear regression analysis: association between MMSE score, sex, education, dentures, and subjective masticatory efficiency.

Dependent Variable	Independent Variables				95% CI for B		*p*
		B	SE B	Beta	Lower Bound	Upper Bound	
MMSE	(Constant)	18.022	3.011		12.051	23.994	0.000
	Sex	1.887	0.511	0.242	0.874	2.899	0.000
	Education	0.815	0.393	0.135	0.036	1.595	0.041
	Edentulism treatment	3.986	1.359	0.517	1.291	6.681	0.004
	Subjective masticatory efficiency	−1.513	1.360	−0.196	−4.210	1.184	0.268

## Data Availability

All the data are available from corresponding authors upon reasonable request.

## References

[1] Fontana L., Kennedy B.K., Longo V.D., Seals D., Melov S. (2014). Medical research: Treat ageing. Nature.

[2] Partridge L., Deelen J., Slagboom P.E. (2018). Facing up to the global challenges of ageing. Nature.

[3] Verma I., Taegen J. (2021). Ageing and Inclusion in Rural Areas. Stud. Health Technol. Inform..

[4] Qi S., Yin P., Wang L., Qu M., Kan G.L., Zhang H., Zhang Q., Xiao Y., Deng Y., Dong Z. (2021). Prevalence of Parkinson’s Disease: A Community-Based Study in China. Mov. Disord..

[5] Etgen T., Sander D., Bickel H., Förstl H. (2011). Mild cognitive impairment and dementia: The importance of modifiable risk factors. Dtsch. Arztebl. Int..

[6] Campisi J., Kapahi P., Lithgow G.J., Melov S., Newman J.C., Verdin E. (2019). From discoveries in ageing research to therapeutics for healthy ageing. Nature.

[7] Slade G., Akinkugbe A., Sanders A. (2014). Projections of U.S. Edentulism Prevalence Following 5 Decades of Decline. J. Dent. Res..

[8] Nyberg L., Wåhlin A. (2020). The many facets of brain ageing. eLife.

[9] Fischl B., Salat D.H., Busa E., Albert M., Dieterich M., Haselgrove C., van der Kouwe A., Killiany R., Kennedy D., Klaveness S. (2002). Whole Brain Segmentation: Automated Labeling of Neuroanatomical Structures in the Human Brain. Neuron.

[10] Voss M.W., Weng T.B., Burzynska A.Z., Wong C.N., Cooke G.E., Clark R., Fanning J., Awick E., Gothe N.P., Olson E.A. (2016). Fitness, but not physical activity, is related to functional integrity of brain networks associated with aging. NeuroImage.

[11] Beydoun M.A., Beydoun H.A., Gamaldo A.A., Teel A., Zonderman A.B., Wang Y. (2014). Epidemiologic studies of modifiable factors associated with cognition and dementia: Systematic review and meta-analysis. BMC Public Health.

[12] Gerstorf D., Herlitz A., Smith J. (2006). Stability of Sex Differences in Cognition in Advanced Old Age: The Role of Education and Attrition. J. Gerontol. B Psychol. Sci. Soc. Sci..

[13] De Bruijn R.F., Akoudad S., Cremers L.G., Hofman A., Niessen W.J., van der Lugt A., Koudstaal P.J., Vernooij M.W., Ikram M.A. (2014). Determinants, MRI Correlates, and Prognosis of Mild Cognitive Impairment: The Rotterdam Study. J. Alzheimer’s Dis..

[14] Fotuhi M., Hachinski V., Whitehouse P.J. (2009). Changing perspectives regarding late-life dementia. Nat. Rev. Neurol..

[15] Reyes-Ortiz C.A., Luque J.S., Eriksson C.K., Soto L. (2013). Self-reported tooth loss and cognitive function: Data from the Hispanic Es-tablished Populations for Epidemiologic Studies of the Elderly (Hispanic EPESE). Colomb. Med..

[16] Henke K. (2010). A model for memory systems based on processing modes rather than consciousness. Nat. Rev. Neurosci..

[17] Tulving E., Markowitsch H.J. (1998). Episodic and declarative memory: Role of the hippocampus. Hippocampus.

[18] Batty G.-D., Li Q., Huxley R., Zoungas S., Taylor B.-A., Neal B., de Galan B., Woodward M., Harrap S.-B., Colagiuri S. (2013). Oral Disease in Relation to Future Risk of Dementia and Cognitive Decline: Prospective Cohort Study Based on the Action in Diabetes and Vascular Disease: Preterax and Diamicron Modified-Release Controlled Evaluation (Advance) Trial. Eur. Psychiatry.

[19] Onozuka M., Watanabe K., Mirbod S.M., Ozono S., Nishiyama K., Karasawa N., Nagatsu I. (1999). Reduced mastication stimulates impairment of spatial memory and degeneration of hippocampal neurons in aged SAMP8 mice. Brain Res..

[20] Makiura T., Ikeda Y., Hirai T., Terasawa H., Hamaue N., Minami M. (2000). Influence of diet and occlusal support on learning memory in rats behavioral and biochemical studies. Res. Commun. Mol. Pathol. Pharmacol..

[21] Jiang Q.-S., Liang Z.-L., Wu M.-J., Feng L., Liu L.-L., Zhang J.-J. (2011). Reduced brain-derived neurotrophic factor expression in cortex and hippocampus involved in the learning and memory deficit in molarless SAMP8 mice. Chin. Med. J..

[22] Terasawa H., Hirai T., Ninomiya T., Ikeda Y., Ishijima T., Yajima T., Hamaue N., Nagase Y., Kang Y., Minami M. (2002). Influence of tooth-loss and concomitant masticatory alterations on cholinergic neurons in rats: Immunohistochemical and biochemical studies. Neurosci. Res..

[23] Watanabe K., Ozono S., Nishiyama K., Saito S., Tonosaki K., Fujita M., Onozuka M. (2002). The molarless condition in aged SAMP8 mice attenuates hippocampal Fos induction linked to water maze performance. Behav. Brain Res..

[24] De Marchi R.J., Hugo F.N., Hilgert J.B., Padilha D.M.P. (2012). Association between number of teeth, edentulism and use of dentures with percentage body fat in south Brazilian community-dwelling older people. Gerodontology.

[25] Peres M.A., Bastos J.L., Watt R.G., Xavier A.J., Barbato P.R., D’Orsi E. (2015). Tooth loss is associated with severe cognitive impairment among older people: Findings from a population-based study in Brazil. Aging Ment. Health.

[26] Hirai T., Koshino H. (2006). Expectations of Prosthetic Dentistry as a Health Science. Nihon Hotetsu Shika Gakkai Zasshi.

[27] Walker K.A., Power M.C., Gottesman R.F. (2017). Defining the Relationship Between Hypertension, Cognitive Decline, and Dementia: A Review. Curr. Hypertens. Rep..

[28] Okamoto N., Morikawa M., Tomioka K., Yanagi M., Amano N., Kurumatani N. (2015). Association between Tooth Loss and the Development of Mild Memory Impairment in the Elderly: The Fujiwara-kyo Study. J. Alzheimer’s Dis..

[29] Folstein M.F., Folstein S.E., McHugh P.R. (1975). “Mini-Mental State”. A Practical Method for Grading the Cognitive State of Patients for the Clinician. J. Psychiatr. Res..

[30] Folstein F.M., Folstein S.E., White T., Messer M.A. (2013). MMSE-2: Mini-Mental State Examination, Ed. A 2-a-Manual de Utilizare a Testului.

[31] Creavin S.T., Wisniewski S., Noel-Storr A.H., Trevelyan C.M., Hampton T., Rayment D., Thom V.M., Nash K.J., Elhamoui H., Milligan R. (2016). Mini-Mental State Examination (MMSE) for the detection of dementia in clinically unevaluated people aged 65 and over in community and primary care populations. Cochrane Database Syst. Rev..

[32] Prince M., Wimo A., Guerchet M., Ali G.C., Wu Y.T., Prina M. (2015). World Alzheimer Report 2015: The Global Impact of Dementia.

[33] Sachdev P.S., Lipnicki D.M., Kochan N.A., Crawford J.D., Thalamuthu A., Andrews G., Brayne C., Matthews F.E., Stephan B.C.M., Lipton R.B. (2015). The Prevalence of Mild Cognitive Impairment in Diverse Geographical and Ethnocultural Regions: The COSMIC Collaboration. PLoS ONE.

[34] Fukushima-Nakayama Y., Ono T., Hayashi M., Inoue M., Wake H., Ono T., Nakashima T. (2017). Reduced Mastication Impairs Memory Function. J. Dent. Res..

[35] Kumar P.S. (2017). From focal sepsis to periodontal medicine: A century of exploring the role of the oral microbiome in systemic disease. J. Physiol..

[36] Peyron M.A., Woda A., Bourdiol P., Hennequin M. (2017). Age-related changes in mastication. J. Oral Rehabil..

[37] Bergdahl M., Habib R., Bergdahl J., Nyberg L., Nilsson L.-G. (2007). Natural teeth and cognitive function in humans. Scand. J. Psychol..

[38] Galindo-Moreno P., Lopez-Chaichio L., Padial-Molina M., Avila-Ortiz G., O’Valle F., Ravida A., Catena A. (2021). The impact of tooth loss on cognitive function. Clin. Oral Investig..

[39] Okamoto N., Morikawa M., Okamoto K., Habu N., Hazaki K., Harano A., Iwamoto J., Tomioka K., Saeki K., Kurumatani N. (2010). Tooth loss is associated with mild memory impairment in the elderly: The Fujiwara-kyo study. Brain Res..

[40] Onozuka M., Fujita M., Watanabe K., Hirano Y., Niwa M., Nishiyama K., Saito S. (2002). Mapping Brain Region Activity during Chewing: A Functional Magnetic Resonance Imaging Study. J. Dent. Res..

[41] Hirano Y., Obata T., Kashikura K., Nonaka H., Tachibana A., Ikehira H., Onozuka M. (2008). Effects of chewing in working memory processing. Neurosci. Lett..

[42] Fontijn-Tekamp F.A., Slagter A.P., Van Der Bilt A., Van ’T Hof M.A., Witter D.J., Kalk W., Jansen J.A. (2000). Biting and chewing in overdentures, full dentures, and natural dentitions. J. Dent. Res..

[43] Ikebe K., Gondo Y., Kamide K., Masui Y., Ishizaki T., Arai Y., Inagaki H., Nakagawa T., Kabayama M., Ryuno H. (2018). Occlusal force is correlated with cognitive function directly as well as indirectly via food intake in community-dwelling older Japanese: From the SONIC study. PLoS ONE.

[44] Lopez-Chaichio L., Padial-Molina M., O’Valle F., Gil-Montoya J.A., Catena A., Galindo-Moreno P. (2021). Oral health and healthy chewing for healthy cognitive ageing: A comprehensive narrative review. Gerodontology.

[45] Cerutti-Kopplin D., Feine J., Padilha D.M., de Souza R.F., Ahmadi M., Rompré P., Booij L., Emami E. (2016). Tooth loss increases the risk of diminished cognitive function: A systematic review and meta-analysis. JDR Clin. Transl. Res..

[46] Okamoto N., Morikawa M., Amano N., Yanagi M., Takasawa S., Kurumatani N. (2017). Effects of Tooth Loss and the Apolipoprotein E ɛ4 Allele on Mild Memory Impairment in the Fujiwara-kyo Study of Japan: A Nested Case-Control Study. J. Alzheimer’s Dis..

[47] Shimazaki Y., Soh I., Saito T., Yamashita Y., Koga T., Miyazaki H., Takehara T. (2001). Influence of dentition status on physical disability, mental impairment and mortality in in-stitutionalized elderly people. J. Dent. Res..

[48] Avivi-Arber L., Sessle B.J. (2018). Jaw sensorimotor control in healthy adults and effects of ageing. J. Oral Rehabil..

[49] Takata Y., Ansai T., Soh I., Akifusa S., Sonoki K., Fujisawa K., Yoshida A., Kagiyama S., Hamasaki T., Nakamichi I. (2008). Relationship between chewing ability and high-level functional capacity in an 80-year-old population in Japan. Gerodontology.

[50] Utsugi C., Miyazono S., Osada K., Matsuda M., Kashiwayanagi M. (2014). Impaired mastication reduced newly generated neurons at the accessory olfactory bulb and pheromonal responses in mice. Arch. Oral Biol..

[51] Kumar A., Kothari M., Grigoriadis A., Trulsson M., Svensson P. (2018). Bite or brain: Implication of sensorimotor regulation and neu-roplasticity in oral rehabilitation procedures. J. Oral Rehabil..

